# Targeting GPC3^high^ cancer-associated fibroblasts sensitizing the PD-1 blockage therapy in gastric cancer

**DOI:** 10.1080/07853890.2023.2189295

**Published:** 2023-04-10

**Authors:** Dinuo Li, Yu Wang, Ce Shi, Shuai Fu, Yi-Fei Sun, Chen Li

**Affiliations:** a Gastric Surgery Department, The First Affiliated Hospital of Jinzhou Medical University, JinZhou, Liaoning, China; b Molecular Testing Center, The First Affiliated Hospital of Jinzhou Medical University, JinZhou, Liaoning, China

**Keywords:** Gastric cancer, cancer-associated fibroblasts, GPC3, immunotherapy, single cell sequencing

## Abstract

Cancer-associated fibroblasts (CAFs) are an important part of tumour microenvironment, but its role in immunotherapy of gastric cancer (GC) is still needed to further study. In this study, we firstly distinguish the GC related CAFs *via* single cell sequencing dataset. CAFs in deep layers of GC tissues gain more developmental potential. Moreover, we found Glypican-3 (GPC3) is up-regulated in the CAFs subgroups of the advanced GC and correlated with poor prognosis in GC patients. In addition, higher GPC3 expression GC patients have higher TIDE (Tumour Immune Dysfunction and Exclusion) score, dysfunction and exclusion score. independent GC cohort also show GC patients with GPC3^high^ CAFs have lower response rate to PD-1 therapy. GPC3 secreted from CAFs up-regulated PD-L1, TIM3, CD24, CYCLIN D1, cMYC and PDK mRNA expression level in HGC-27 cells. At last, *in vivo* model demonstrate that targeting GPC3^high^ CAFs sensitizing the PD-1 blockage therapy in GC. In conclusion, GPC3 expression in CAFs is a critical prognostic biomarker, and targeting GPC3^high^ cancer-associated fibroblasts sensitizing the PD-1 blockage therapy in GC.

**Key messages**
1. Glypican-3 (GPC3) is up-regulated in the CAFs subgroups of the advanced gastric cancer.
2. Gastric cancer patients with GPC3^high^ CAFs have lower response rate to PD-1 therapy.
3. Targeting GPC3^high^ CAFs sensitizing the PD-1 blockage therapy in gastric cancer.

Gastric cancer (GC) is the fifth most common malignant tumour in the world, ranking the third leading cause of malignant tumour death in the world, with about 780,000 deaths every year [1]. In recent years, with the development of medical technology and the popularization of knowledge of prevention and treatment of GC, the diagnosis rate, survival time and quality of life of early GC in the world had been significantly improved [2]. However, 2/3 of the patients are still in the advanced stage at the time of initial treatment, and the five-year survival rate is still lower than 30% even if they receive comprehensive treatment based on surgery [3]. The treatment status of patients with advanced GC is not optimistic, the curative effect of chemotherapy has reached the bottleneck, and the clinical practice of targeted therapy in GC is not successful, resulting in limited treatment options for GC at present [4]. In recent years, the emergence of immunotherapy has brought hope to the treatment of advanced GC [5]. The combination therapy of programmed death receptor-1 (PD-1) and programmed death ligand (PD-L1) inhibitors at immune checkpoint has achieved good curative effect in the treatment of GC [6]. However, Immunotherapy for advanced GC faces certain difficulties. Compared with malignant tumours with strong immunity, GC has limited benefits from immunotherapy [7]. How to screen the best beneficiaries is crucial. Therefore, exploring more immunotherapy schemes and finding effective immunotherapy biomarkers will provide new therapeutic strategies for immunotherapy of GC.

Cancer-associated fibroblasts (CAFs) are an important part of tumour microenvironment, which are closely related to tumour growth, invasion, metastasis and curative effect [8]. CAFs can be derived from local normal fibroblasts, bone marrow mesenchymal stem cells (MSCs), epithelial cells, and can also be transformed from myofibroblasts, local pericytes, fibroblasts and even adipocytes [9]. According to the different roles of fibroblasts in tumours, CAFs can be divided into five types [10]: F1 (anti-tumour CAFs), F2 (tumour-promoting CAFs), F3 (secretory CAFs), F4 (ECM remodelling CAFs) and F5 (other functional CAFs). CAFs also plays an important role in the formation of immunosuppressive tumour microenvironment (TME). CAFs helps recruit monocytes to differentiate into M2 macrophages, which may affect PD-1 pathway to perform immunosuppressive function [11]. In the pancreatic cancer model, FAP-positive CAFs can secrete CXCL12, enhance STAT3-CCL2 signal transduction, enhance MDSC recruitment, promote the production of regulatory T cells and tumour-associated macrophages, and promote immunosuppression [12]. However, as for whether targeting CAFs can enhance the immunotherapy effect of GC patients, further research is still needed to confirm.

Gypican-3 (GPC3) has been recognized as an oncofoetal protein in hepatic neoplasms and yolk sac tumours [13]. Many studies had reported that GPC3 can interact with various growth factors, signal molecules and participate in many different signal pathways. At the same time, it will be influenced by various other biological factors in tumour cell microenvironment, which will affect the proliferation, migration, invasion and development of liver cancer in different angles and degrees [14,15]. For GC, previous study report GPC3 expression is markedly decreased and may play a tumour suppressor role in GC [16]. Downregulating GPC3 in GC can inhibit gastric cancer cell metastasis and impact the tumour immune microenvironment [17]. Therefore, the role of GPC3 in the GC is still controversial.

In this study, we firstly distinguish the GC related CAFs *via* single cell sequencing dataset. Moreover, we found GPC3 is up-regulated in the CAFs subgroups of the advanced GC and correlated with poor prognosis. In addition, we also found GC with GPC3^high^ CAFs is correlated with lower response proportion for PD-1 blockage. At last, *in vivo* model demonstrate that targeting GPC3^high^ CAFs sensitizing the PD-1 blockage therapy in GC. Herein, we found GPC3 is a critical biomarker and therapeutic target for sensitizing the PD-1 blockage therapy in GC.

## Materials and Methods

### Single-cell RNA-seq extraction and data processing

The single-cell data used for this research were downloaded from online public database, which present no ethical issues. We obtained processed single-cell gastric cancer data from GEO data sets (GSE167297 including 30,365 cells of 14 samples from five patients.

The Seurat R package was primarily applied for quality control procedures and downstream bioinformatic analyses. We first filtered out cells with low quality that fit any of the following criteria: the proportion of mitochondrial genes counts (>20%), nCount_RNA< 500 or nCount_RNA> 50,000, nFeature_RNA < 200 or nFeature_RNA > 4000. The scDblFinder package was utilized to remove the potential doublets with the default settings. After these quality control procedures, we conducted a series of preprocessing procedures for downstream analysis for the remaining 21,406 cells. In detail, we employed a global-scaling normalization method LogNormalize that normalized the feature expression for each cell by the total expression and multiplied this by a scale factor (10,000 by default), and log-transformed the result using the NormalizeData() function in Seurat. The top 3000 highly variable genes (HVGs) from the normalized expression matrix were identified before we performed the principal component analysis (PCA) based on these HVGs. The batch effects were removed by the Harmony package of R based on the top 50 PCA components identified.

The PCElbowPlot function in Seurat was utilized to select the optimal number of PCs for further analysis as recommended by Seurat. The FindNeighbors and FindClusters functions in Seurat were both applied for cell clustering. The RunUMAP function were performed for visualization when appropriate. The cell identity of each cluster was defined based on the expression of known marker genes. We identified B cells (CD79A, MS4A1), T cells (CD3D, CD3E), Monocytes (CD68, CD14), Plasma cells (MZB1, SDC1), Epithelial cells (EPCAM, KRT19), Endothelial cells (TM4SF1), Fibroblasts(COL1A1,FGF7), Mast cells(TPSAB1,CPA3), Multipotent progenitor cells(UBE2S, H2AFX), Multilymphoid progenitor cells(COTL1, TMSB10) and Mesenchymal cells(ACTA2, AKAP12). Due to the transcriptional similarities between NK and T cells, we conducted the second round of clustering to distinguish T and NK cells. After that, we conducted the second round of high-resolution clustering to identify the finer subclusters within Fibroblasts. Procedures of the second round of clustering were identical to the first one.

### Inference of cell state by trajectory analysis

To predict the relative differentiation state of cells, we performed Cytotrace analysis based on the expression data in Fibroblasts sub-clustering results. And the trajectory analysis was performed using the Monocle3 package to reveal the cell-state transitions. The DEGs changed along with the pseudotime were identified using the graph_test function in Monocle2.

### Analysis of bulk RNA-seq data

The bulk RNA-seq profiles were download from the TCGA dataset(http://xena.ucsc.edu/).The gene-level expression of each sample was calculated by aggregating transcript expression (Fragments Per Kilobase of exon model per Million mapped fragments, FPKM) belonging to the same gene. The corresponding clinical data was also downloaded. Kaplan–Meier (KM) analysis was undertaken using R package survival.

TIDE (http://tide.dfci.harvard.edu/) is a computational method that integrates the expression signatures of T cell dysfunction and exclusion to model tumour immune evasion. We used the TIDE algorithm to predict the clinical response to immune checkpoint blockade (ICB) in GC patients based on pretreatment genomics.

### Clinical samples and immunofluorescence

50 copies of GC tissue and paired normal tissue were obtained from the First Affiliated Hospital of Jinzhou Medical University. All patients received surgery and have recurrence, using the chemotherapy combined with immunotherapy. Another 15 fresh GC tissues were used to analyse the correlation between GPC3 expression and tumour infiltrating CD8+ T cells. This study was approved by the Ethics Committee of the First Affiliated Hospital of Jinzhou Medical University (reference number KY 2022-022-07) and conducted in accordance with the Declaration of Helsinki.

### Cell culture and transfection

Mouse GC cell line (YTN16) was described previously. Mice L929 cell line (mouse fibroblast) purchased from the ATCC. Human skin fibroblasts and HGC-27 gastric cancer cell line were purchased from IMMOCELL company. These four cell lines were cultured in 90% Roswell Park Memorial Institute (RPMI) 1640 medium, with 10% foetal bovine serum (FBS) and 1% streptomycin and penicillin at 37 °C in a 5% CO^2^ cell incubator. Mouse and human GPC3-overexpressing (GPC3-OE) lentivirus was purchased from Invabio (Shanghai, China). GPC3-overexpressing lentivirus (1 × 10^8^ TU/ml) and polybrene (8 μg/ml) were added into L929 cells and cultured for 72 h. The transfected cells were screened using puromycin (1 μg/ml). L929 cells successfully overexpressing GPC3 were detected by immunofluorescence. The control group was transfected by lentivirus with empty vector.

### Mouse subcutaneous xenograft model

C57bl/6 mice were provided by the Cyagen company. The mice were subcutaneously co-inoculated with YTN16 and L929 cells with 1:1 proportion. L929 cells with vector or GPC3-OE were used to establish xenograft model. Tumours were allowed to grow for 10 days after inoculation with tumour cells and fibroblast. After the tumour grow, mice were randomly divided into four groups, each group with 5 mice. The treatment groups were as follows: (1) vector group: treat with isotype antibody; (2) vector + anti-PD-1 group: mice was subcutaneous injected with a PD-1 inhibitor (anti-mouse PD-1 monoclonal antibody, Clone RMP1-14, BioXcell), 5 mg/kg every three days for a total of five times; (3) GPC3 group: YTN16 cells and L929 (GPC3-OE) were co-inoculated to establish xenograft model; (4) GPC3 + PD-1 inhibitor group: YTN16 cells and L929 (GPC3-OE) were co-inoculated to establish xenograft model, and treat with PD-1 inhibitor. Moreover, to detect the combined effect with anti-GPC3 and anti-PD-1, YTN16 cells and L929 (GPC3-OE) were co-inoculated to establish xenograft mode, and the treatment groups were as follows: (1) IgG group: mice was treated with isotype antibody; (2) anti-PD-1 group: PD-1 inhibitor was subcutaneous injected 5 mg/kg every three days for a total of five times. (3) anti-GPC3 group: mice was treated with mouse anti-GPC3 recombinant antibody (CBMAB-G4509-LY, Creativebiolabs), 5 mg/kg every three days for a total of five times. (4) anti-GPC3 + anti-PD-1 group: mice was treated anti-GPC3 recombinant antibody and PD-1 inhibitor.

### Immunofluorescence

All tissues are examined pathologically by two pathologists. Serialized sects (4 microns) is cut from paraffin-embedded GC tissues or mice tumour tissues. All sections are dewaxed, hydrated, endogenous enzyme removal, and antigen retrieval. Subsequently, Anti-GPC3 antibody(1:500; Abcam, ab216606, ab181150), Anti-αSMA antibody (1:200; Abcam, ab124964, ab240654), and related fluorescent secondary antibody (ThermoFisher Scientific). To determine the expression of GPC3, we calculated the mean integral optical density (IOD) per slice using Image-Pro Plus 6.0 software (Media Cybernetics, USA).

### Isolation of tissue T cells

The fresh human tissue or mice tissue were separated by operation from patient or mice. The tumour tissue was sieved through a cell sieve, and crushed. After the supernatant was removed, red blood cell lysates were added and the supernatant was removed following centrifugation. The cells were cultured and resuspended to remove insoluble tissue fibres, and the cells in the suspension were counted. Then, CD8+ T cells were isolated using the EasySep™ human T-cell enrichment kit and EasySep™ mouse T-cell enrichment kit (Stem Cell Technologies).

### Flow cytometry

The density of infiltrating CD4+ T and CD8+ T cells in the tumours and the expression levels of interferon (IFN)-γ were evaluated using flow cytometry. Fresh GC tumour tissue and mice subcutaneous xenograft tissue samples were taken to obtain tumour-infiltrating lymphocytes. Tumour-infiltrating lymphocytes were suspended, and 1 μl antibodies were added into the tube. Antibodies included Fluorescein isothiocyanate (FITC) anti-human CD8 (980908, BioLegend), FITC Mouse Anti-Human CD4 (550628, BD Biosciences), and PE anti-human IFN-γ Antibody (383303 , BioLegend), FITC anti-mouse CD8a Antibody PE (100705, BioLegend), anti-mouse IFN-γ Antibody (505807, BioLegend). After routine operation, the supernatants were collected and flow cytometry was performed.

### RT-qPCR

Total RNA was extracted from cells with TRIzol reagents (Invitrogen, USA). cDNA is generated according to the protocol of the manufacturer’s kit (Takara). With an Applied Biosystem instrument with a reaction volume of 20 l (SYBR Green Real-Time PCR Master Mix, Qingdao, Beijing, China). The gene GAPDH is used as an internal parameter. All samples were tested three times. And qRT-PCR primers for this study are listed in Table 1.

**Table 1.** The qRT-PCR primers for this study.

Gene		Primer
PD-L1	Forward	5′-TGGCATTTGCTGAACGCATTT-3′
	Reversed	5′-TGCAGCCAGGTCTAATTGTTTT-3′
TIM3	Forward	5′-CTGCTGCTACTACTTACAAGGTC-3′
	Reversed	5′-GCAGGGCAGATAGGCATTCT-3′
CD24	Forward	5′-CTCCTACCCACGCAGATTTATTC-3′
	Reversed	5′-AGAGTGAGACCACGAAGAGAC-3′
CYCLIN D1	Forward	5′-GCTGCGAAGTGGAAACCATC-3′
	Reversed	5′-CCTCCTTCTGCACACATTTGAA-3′
	Forward	5′-GGCTCCTGGCAAAAGGTCA-3′
	Reversed	5′-CTGCGTAGTTGTGCTGATGT-3′
PDK	Forward	5′-CTGTGATACGGATCAGAAACCG-3′
	Reversed	5′-TCCACCAAACAATAAAGAGTGCT-3′
GAPDH	Forward	5′-TCGGAGTCAACGGATTTGGT-3′
	Reversed	5′-TTCCCGTTCTCAGCCTTGAC-3′

### Statistical analysis

The data are presented as mean ± SD after analysis using GraphPad Prism 7.0 (GraphPad, San Diego, CA). Differences between two groups were evaluated with two-tailed unpaired t-test, and differences among more than two groups were evaluated using one-way ANOVA followed by Tukey’s post hoc test. *p* < .05 indicated a significant difference. Survival curves were plotted using the KM method and compared using the log-rank test.

## Results

### Single cell sequencing identify CAFs is increased in the advanced GC

**Figure 1.**
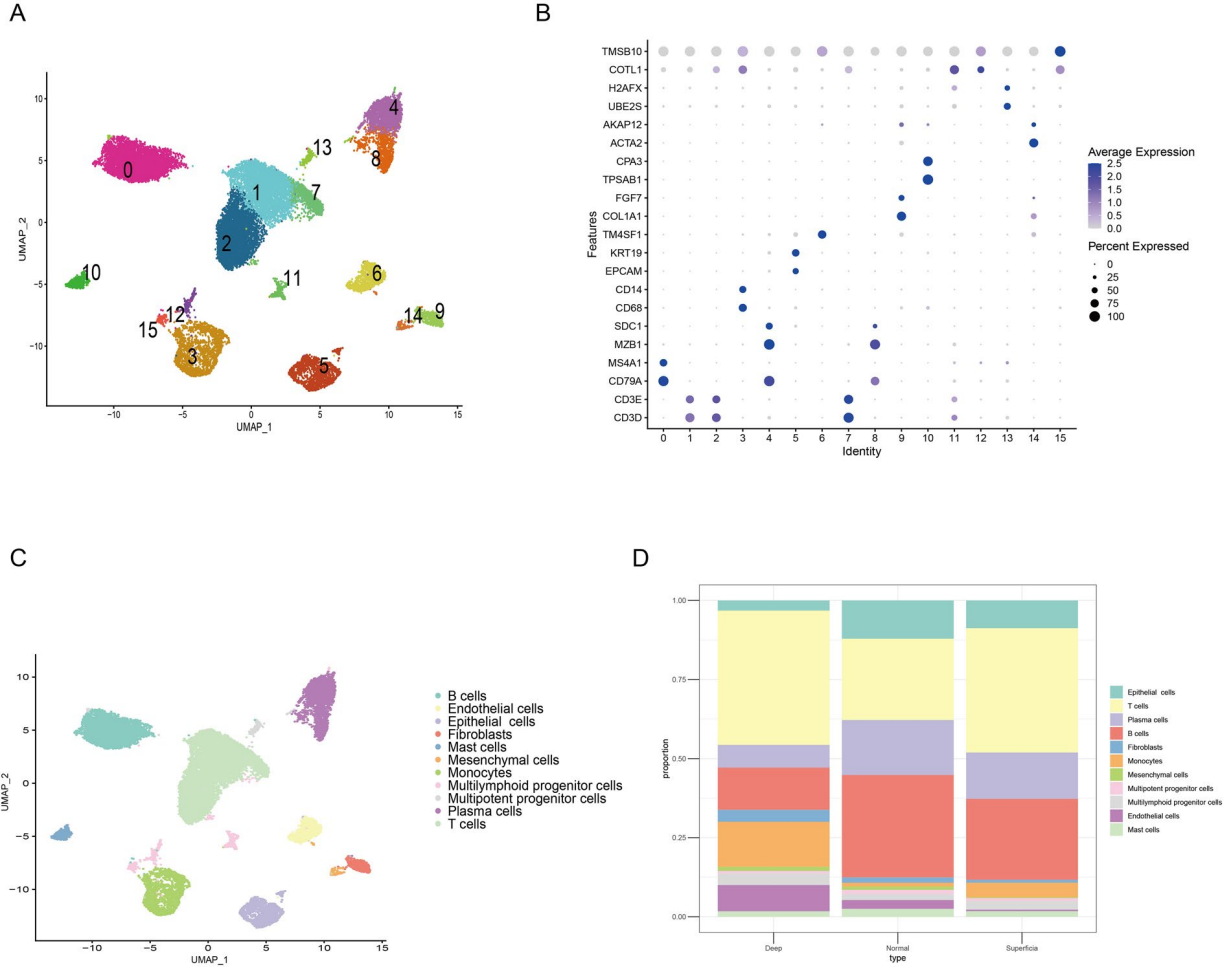
Single cell sequencing identify CAFs is increased in the advanced GC. (A) UMAP map consisting of 23,060 cells in 15 clusters from five patients with diffuse-type gastric cancer. (B) Visualization of the marker genes for each cluster of cells. (C) Annotation of cells from gastric cancer and normal tissues showing a total of 15 clusters of cells. (D) The proportion of the 15 clusters in normal, superficial and deep layers of tumours, the cell number of fibroblasts was increased in deep layers of tumour compared with the normal or superficial tumour tissue.

To explore the tumour microenvironment difference between the early and advanced gastric cancer, we use a published single cell sequencing dataset (GSE167297) to analyse the heterogeneity of GC. A total of five patients with diffuse-type GC were using to single cell sequencing analysis, and 23,060 cells in 15 clusters can be identified in the all samples (Figure 1(A)). Moreover, to clarify cell types of 15 clusters, the different marker gene of the 15 clusters was used to definite the cell types (Figure 1(B)). Base on the marker genes express level, a total of 11 types of cells were identified, included B cells, endothelial cells, epithelial cells, fibroblast, mast cells, mesenchymal cells, Monocytes, multilymphoid progenitor cells, plasma cells and T cells (Figure 1(C)). As the depth of tumour invasion is the critical factor for the staging of GC, we found the deep layers of GC tissues has an increased fibroblasts compared with the normal gastric tissues and superficial GC tissues (Figure 1(D)). Thus, these single cell sequencing data showed the increased CAFs in the advanced GC.

### CAFs in deep layers of GC tissues gain more developmental potential

**Figure 2.**
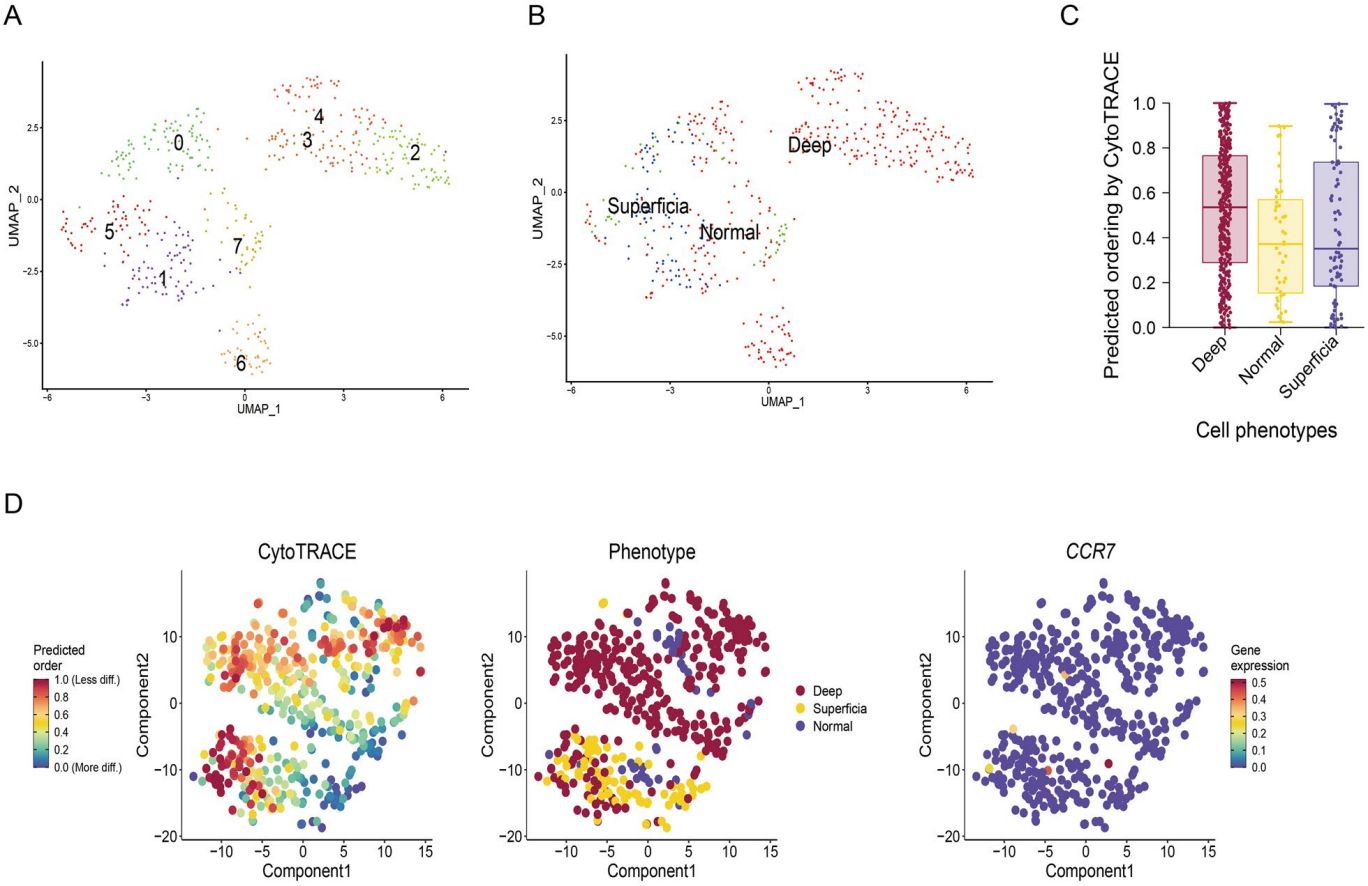
Developmental trajectories of fibroblasts in GC. (A) UMAP map was used to analyse the subgroups of fibroblasts. (B) The clustering of cells from normal gastric tissue, superficial and deep layers of tumour tissue. (C) CytoTRACE software is used to evaluate the developmental potential of fibroblasts. (D) Developmental potential comparison of fibroblasts in different tissues.

To further clear the heterogeneity and developmental trajectory, the UMAP map was used to analyse the subgroups of fibroblasts. We found seven clusters of fibroblasts can be found in the all samples (Figure 2(A,B)). And the clustering of cells from normal gastric tissue, CytoTRACE software is used to evaluate the developmental potential of fibroblasts, we found the fibroblasts in deep layers of tumour tissue has a higher predicted ordering compared with normal gastric tissue and superficial layers of tumour tissue (Figure 2(C,D)). These result showed that CAFs in deep layers of GC tissues gain more developmental potential.

### GPC3 is up-regulated in the CAFs of the advanced GC

**Figure 3.**
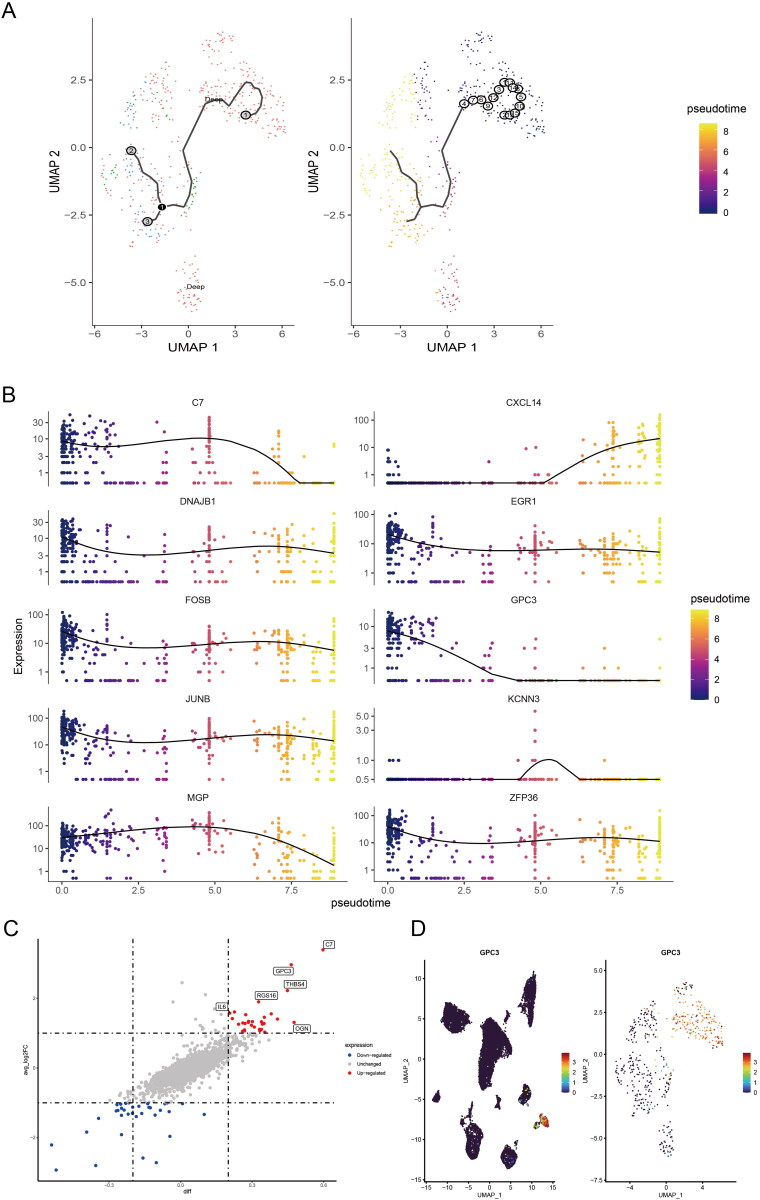
GPC3 is up-regulated in the CAFs of the advanced GC. (A) UMAP map showed single-cell trajectory of CAFs in normal gastric tissues, superficial and deep layers of tumours by pseudotime analysis. (B) The expression level of different expression genes, which included C7, CXCL14, DNAJB1, EGR1, FOSB, GPC3, JUNB, KCNN3, MGP, ZFP36 in the different clusters of CAFs in normal gastric tissues, superficial and deep layers of tumours by pseudotime analysis. (C) The scatter diagram showed the different expression genes of CAFs in superficial and deep layers of tumours. (D) The GPC3 expression level in the 15 clusters and the different clusters of CAFs.

In order to make clear the changes of cell trajectory of CAFs in different depth of layers of GC tissues. We performed the pseudotime analysis for trajectory change of fibroblast clusters of the all samples. The UMAP map showed single-cell trajectory of CAFs in all samples, and with the depth of tumour invasion in GC, we can find fibroblasts have a changing trajectory from normal gastric or superficial GC tissues to deep layers of GC tissues (Figure 3(A)). Moreover, we found C7, CXCL14, DNAJB1, EGR1, FOSB, GPC3, JUNB, KCNN3, MGP, ZFP36 are involved the changing trajectory of fibroblasts from normal gastric or superficial GC tissues to deep layers of GC tissues (Figure 3(B)). The different expression genes between deep layers of GC tissues and normal gastric or superficial GC tissues was shown in Figure 3(C). Interesting, Glypican-3 (GPC3) was mainly high expression in the fibroblasts of GC, as well as mainly up-regulated in the deep layers clusters of fibroblasts in GC tissues (Figure 3(D)).

### GC with GPC3^high^ CAFs is correlated with poor prognosis

**Figure 4.**
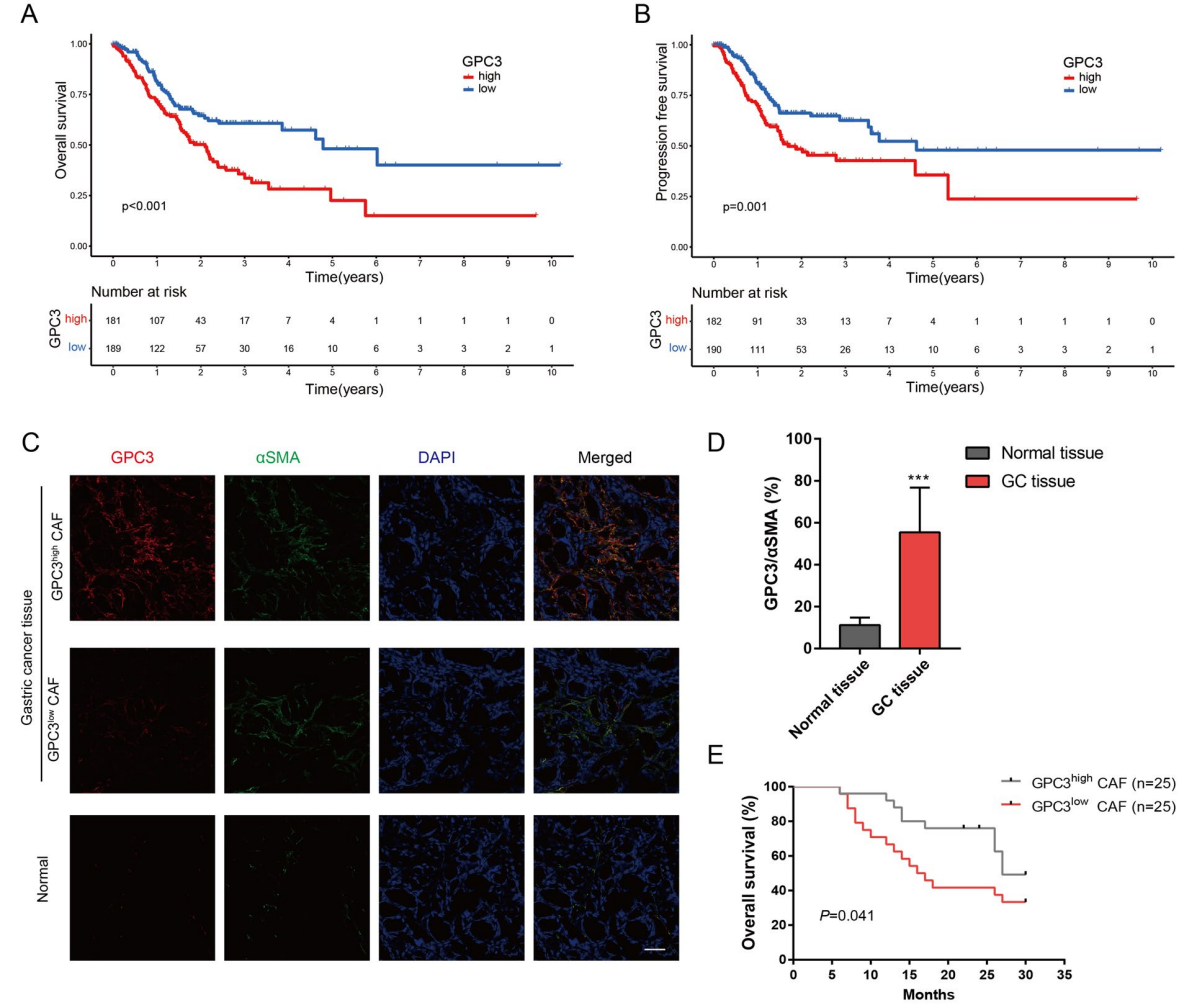
GC with GPC3^high^ CAFs is correlated with poor prognosis. (A,B) Kaplan–Meier survival curve shows the relationship between high- and low-GPC3 expression and GC prognosis with TCGA dataset. (C) Immunofluorescence were used to detect the GPC3 expression in CAFs of GC tissue and normal tissue (bar = 50 μm). (D) Comparison between the GPC3 expression level in CAFs of GC tissue and normal tissue. (E) Kaplan–Meier survival curve shows the relationship between high- and low-GPC3 expression in CAFs and OS of GC patients (***p < .001).

To further explore the relationship between GPC3 and clinical features of GC, we firstly compared with the prognosis of GC patients with different GPC3 expression level. High GPC3 expression in GC tissues correlated with higher TNM stage (Table 2), poor overall survival (OS) and progression free survival (PFS) in GC patients (Figure 4(A,B)). Moreover, we also detect the GPC3 expression in GC or normal gastric tissue *via* immunofluorescence. We found GPC3 mainly expression in CAFs in GC tissue (Figure 4(C)), and GPC3 in GC tissue also higher expression compared with the normal gastric tissue (Figure 4(D)). And high GPC3 expression in CAFs of GC correlated with more advanced stages in GC patients (Figure 4(F)). At last, our independent GC cohort also show high GPC3 expression in CAFs correlated with poor OS of patients. Therefore, these result reveal GC with GPC3^high^ CAFs is correlated with poor prognosis.

**Table 2.** Correlations between CAF GPC3 expression and clinicopathological characteristics in GC.

Parameters	No	CAF GPC3 expression		*p* Value
		Low (*n* = 25)	High (*n* = 25)	
Age (years)				.265
<60	27	14	11	
≥60	23	9	14	
Gender				.561
Male	19	8	11	
Female	31	17	14	
Differentiation grade				.769
Well/moderate	18	8	10	
Poor/undifferentiated	32	17	15	
Tumour size (cm)				.778
<5	24	13	11	
≥5	26	12	14	
TNM stage				.021
I/II	29	19	10	
III/IV	21	6	15	
CPS score				.762
<5	34	18	16	
≥5	16	7	9	
MSI/MMR				.417
pMMR/MSI-L/MSS	43	23	20	
dMMR/MSI-H	7	2	5	

### GC with GPC3^high^ CAFs is correlated with lower response of PD-1 blockage therapy

**Figure 5.**
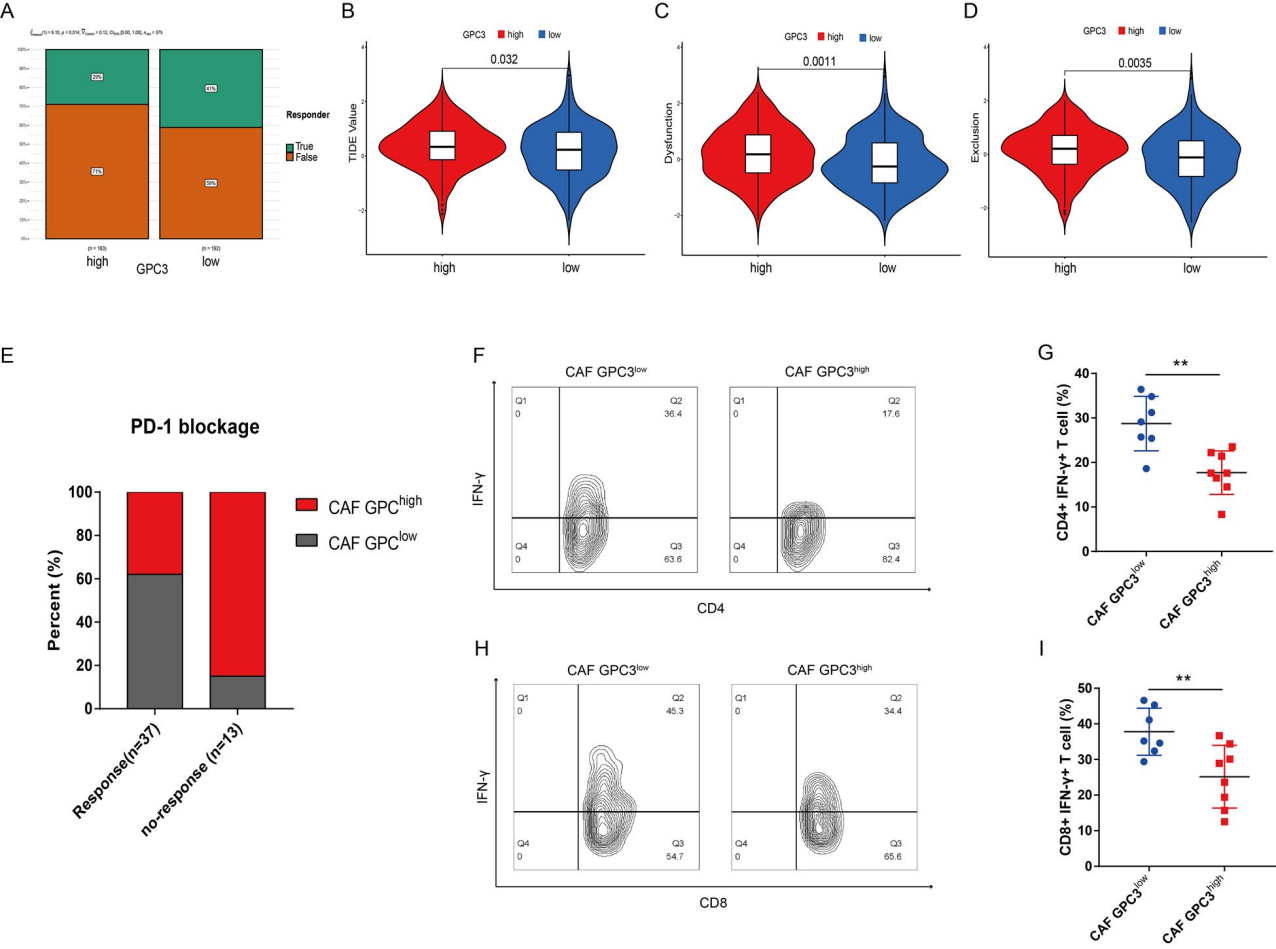
GC with GPC3^high^ CAFs is correlated with lower response of PD-1 blockage therapy. (A) The likelihood of the clinical response to anti-PD-1 therapy for high and low GPC3 expression GC patients from the TCGA cohorts. True represents immunotherapy responders, while false represents immunotherapy nonresponders. (B–D) The violin plots present of TIDE value, immune dysfunction and immune exclusion in high and low GPC3 expression GC patients. (E) The relationship between different GPC3 expression in CAFs and the response ratio of anti-PD-1 therapy in GC patients. (F–I) The proportion of interferon (IFN)-γ-positive cells of CD4+ or CD8+ T cells in the tumours with different GPC3 expression in CAFs.

To further study the relationship between GPC3 and immunotherapy in GC patients, GC patients with higher GPC3 expression has lower response rate to PD-1/PD-L1 therapy (Figure 5(A)). And higher GPC3 expression GC patients have higher TIDE (Tumour Immune Dysfunction and Exclusion) score, dysfunction and exclusion score (Figure 5(B–D)). Moreover, our independent GC cohort also show GC patients with GPC3^high^ CAFs have lower response rate to PD-1 therapy (Figure 5(E)). In addition, GC patients with GPC3^high^ CAFs have lower ratio CD4+ IFN-γ+ and CD8+ IFN-γ+ T cells (Figure 5(F–I)). Therefore, GC patients with GPC3^high^ CAFs closely correlated with the effect of PD-1 blockage therapy and the activity of tumour infiltrated CD4+ and CD8+ T cells.

### GPC3^high^ CAFs impact the effect of PD-1 blockage therapy in GC

**Figure 6.**
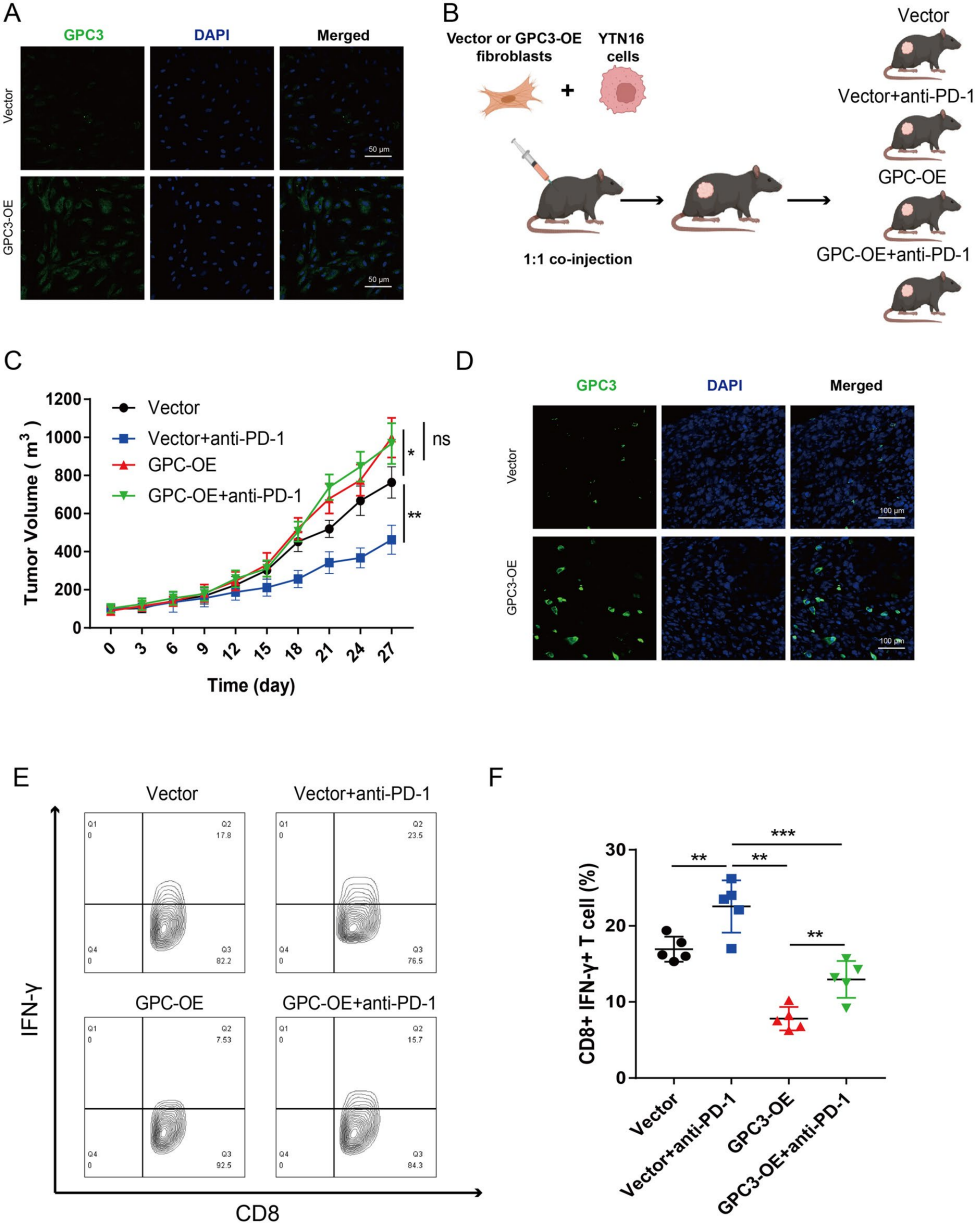
GC with GPC3^high^ CAFs is insensitive to PD-1 blockage therapy *in vivo*. (A) The mice fibroblasts (L929 cells) after GPC3 overexpression lentivirus transfection. (B) The mice xenograft model construction and divide into four groups. (C) Tumour volume comparison after different treatment in the mice GC xenograft model. (D) Immunofluorescence were used to detect the GPC3 positive CAFs in xenograft tumour tissue. (E,F) The proportion of interferon (IFN)-γ-positive cells of CD8+ T cells in the tumours of different groups (*p < .05; **p < .01, ***p < .001).

To further confirm the relationship between GPC3 expression CAFs and immunotherapy in GC. We construct a high GPC3 expression mice fibroblasts cell lines (Figure 6(A)), then fibroblasts cells with high GPC3 expression and YTN16 cell were co-injection to establish a mice xenograft model (Figure 6(B)). As expected, GC tumour with high GPC3 expression fibroblasts is insensitive to PD-1 blockage therapy (Figure 6(C,D)). And GC tumour with GPC3^high^ CAFs also have lower ratio CD8+ IFN-γ+ T cells (Figure 6(E,F)). Therefore, these results showed GC with GPC3^high^ CAFs is insensitive to PD-1 blockage therapy *in vivo*.

### Targeting GPC3 sensitizing the PD-1 blockage Therapy in GC

**Figure 7.**
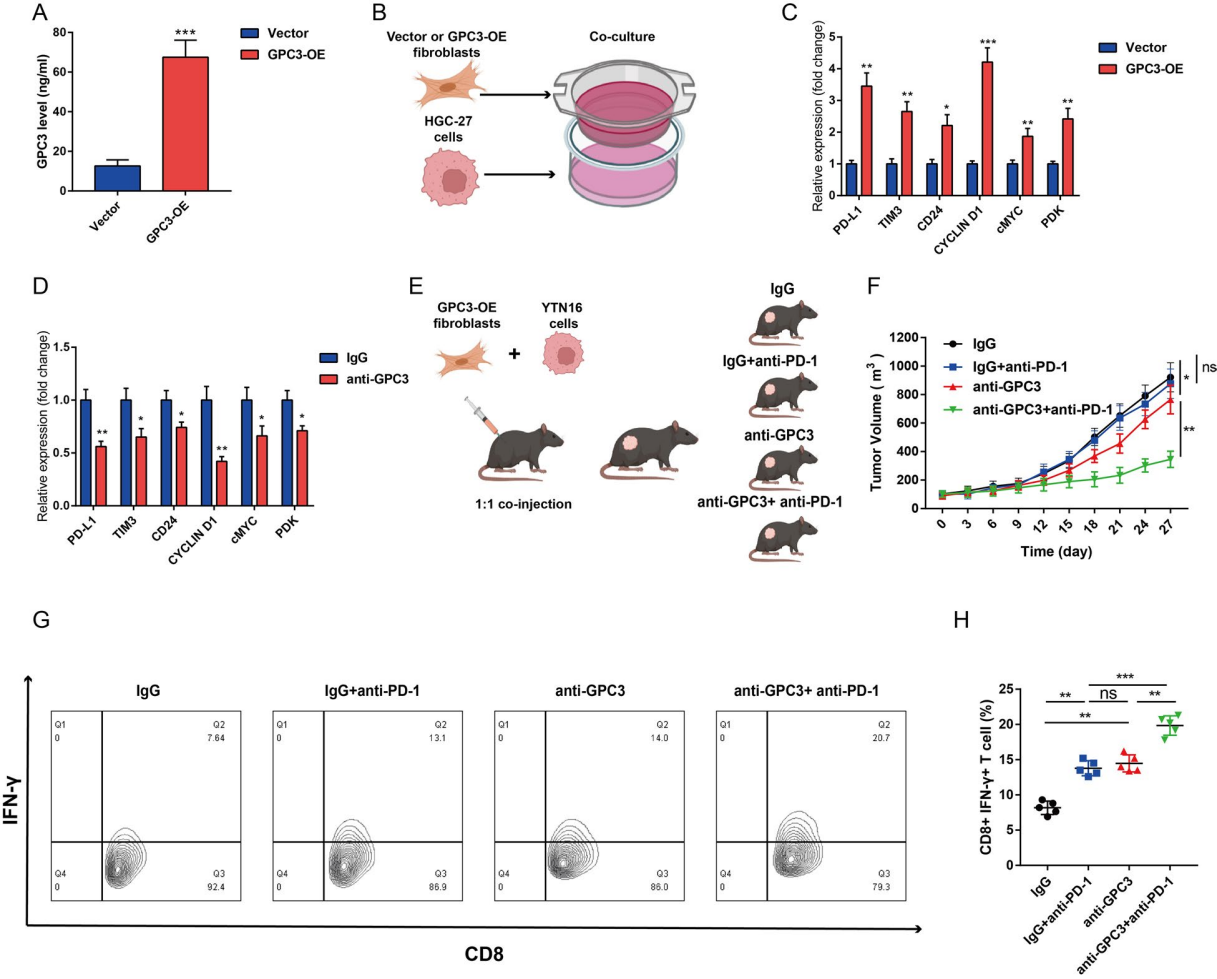
Targeting GPC3 sensitizing the PD-1 blockage therapy in GC. (A) ELISA was used to detect the culture medium after GPC3 overexpression in mice fibroblasts. (B) The co-culture model to study the impact fibroblasts with GPC3 overexpression of to HGC-27 cells. (C) rt-qPCR was used to detect the immune checkpoints expression after co-culture with fibroblasts, the immune checkpoint genes included PD-L1, TIM3, CD24, CYCLIN D1, cMYC and PDK. (D) The immune checkpoint genes expression of HGC-27 cells under the anti-GPC3 treatment. (E) The mice xenograft model construction and divide into four groups. (F) Tumour volume comparison after different treatment in the mice GC xenograft model. (G,H) The proportion of interferon (IFN)-γ-positive cells of CD8+ T cells in the tumours of different groups (*p < .05; **p < .01, ***p < .001).

At last, to further explore the role of GPC3 in CAFs, we found fibroblasts with high GPC3 expression has a higher GPC3 level in culture medium (Figure 7(A)). Then we co-cultured the fibroblasts and HGC-27 cells, the PD-L1, TIM3, CD24, CYCLIN D1, cMYC and PDK mRNA expression level in HGC-27 cells were increased (Figure 7(B,C)). Targeting GPC3 with GPC3 neutralizing antibody in the co-culture system significantly reduce the PD-L1, TIM3, CD24, CYCLIN D1, cMYC and PDK expression caused by fibroblasts (Figure 7(D)). Moreover, Targeting GPC3 *via* GPC3 neutralizing antibody also sensitize the PD-1 blockage therapy *in vivo* (Figure 7(E,F)). And the combined using of anti-GPC3 and anti-PD-1 treatment significantly increased the tumour infiltrated CD8+ IFN-γ+ T cells (Figure 7(G,H)). Therefore, our results further confirmed that targeting GPC3 sensitizing the PD-1 blockage Therapy in GC.

## Discussion

Gastric cancer has a special tumour microenvironment, which promotes the development and metastasis of gastric cancer. Gastric cancer cells form a dynamic network with extracellular matrix (ECM), stromal cells, immune cells and inflammatory cells [18]. By releasing a variety of cytokines, they communicate with each other under the action of various signal pathways. This dynamic network can recruit and transform cells, thus forming a microenvironment that supports tumour growth [19]. Among them, CAFs play an essential role in the occurrence and development of gastric cancer, because they are involved in the release of a variety of cytokines and are widely associated with inflammatory cells, nerve cells and vascular endothelial cells [20]. As the heterogeneity, the role of CAFs in GC is still not completely clear. In this present study, we clarified the heterogeneity of CAFs in GC, and firstly found GPC3 is up-regulated in the CAFs of the advanced GC. Moreover, GC with GPC3^high^ CAFs is correlated with poor prognosis and correlated with lower response of PD-1 blockage therapy. Targeting GPC3 sensitize the PD-1 blockage therapy in GC and significantly increased the tumour infiltrated CD8+ IFN-γ+ T cells.

GPC3 is a glycoprotein on cell membrane, and its amino terminal is a soluble protein, which can be secreted into peripheral blood [21]. GPC 3 is highly expressed in the liver and kidney in embryonic stage, but hardly expressed in normal adult tissues [22]. It is highly expressed in hepatocellular carcinoma, squamous cell carcinoma of lung, clear cell carcinoma of ovary and melanoma. GPC3 can promote the proliferation and tumour formation of hepatocellular carcinoma cells [23–25]. Previously, GPC3 targeted immunotherapy strategy using antibody or peptide vaccine has been developed for the treatment of hepatocellular carcinoma [26]. Meanwhile, serum GPC3 is a biomarker for early diagnosis, prediction of recurrence and evaluation of anti-GPC3 treatment response [27,28]. In our study, we found GPC3 is mainly up-regulated in CAFs in GC tissue, especially for the deep layer of GC, which means GPC3^high^ CAFs is correlated with the tumour progression of GC. Our result also show the GPC3 expression in CAFs can be a potential marker for prognosis of GC.

Moreover, we also found the GPC3 also can be secreted from the CAFs. This also confirm the previous studies for that GPC3 is also up-regulated in the serum of hepatocellular carcinoma, which imply that GPC3 can be a biomarker for effective diagnosis of hepatocellular carcinoma. Our study further found the GC cells can be regulated by the crosstalk mechanism by the GPC3 secretion. GPC3 can regulated the immune check point genes expression, and blockage the GPC3 also can rescue the effect. Although GPC3 peptide vaccines had been developed for inducing GPC3-specific cytotoxic T cells in most vaccinated patients and thereby improve their prognosis [29]. However, the relationship between GPC3 and the response of PD-1 therapy is still not clear. Our study also firstly found GPC3^high^ CAFs is correlated with lower response of PD-1 blockage therapy in GC. Previous study report XCL1/GPC3 fusion gene immunization generates potent antitumour cellular immunity and enhances Anti–PD-1 efficacy [30], but our study further confirm targeting GPC3 can sensitize the PD-1 blockage therapy in GC. As the Nivolumab combined chemotherapy has been approved for the first-line treatment of advanced gastric cancer [6], but further develop more combined treatment strategy can be improve the immunotherapy in GC. Our study reveal that combining anti-GPC3 and anti-PD-1 blockage is an effective treatment strategy in GC. But more basic research and further clinical trials are still need to explore the underlying mechanism and effect of combining anti-GPC3 in immunotherapy in GC.

In conclusion, GPC3 is up-regulated in the CAFs of the advanced GC. Moreover, GC with GPC3^high^ CAFs is correlated with poor prognosis and correlated with lower response of PD-1 blockage therapy. GC with GPC3^high^ CAFs is insensitive to PD-1 blockage therapy in GC. Targeting GPC3 sensitize the PD-1 blockage therapy in GC and significantly increased the tumour infiltrated CD8+ IFN-γ+ T cells.

## Data Availability

The original contributions presented in the study are included in the article. Further inquiries can be directed to the corresponding author.
